# Surgical resections of ulcerative colitis associated with dysplasia or carcinoma

**DOI:** 10.1186/s12957-015-0499-4

**Published:** 2015-02-21

**Authors:** Shinichi Sameshima, Shinichiro Koketsu, Emiko Takeshita, Yawara Kubota, Takashi Okuyama, Kazuyuki Saito, Yoshihiko Ueda, Toshio Sawada, Masatoshi Oya

**Affiliations:** Department of Surgery, Dokkyo Medical University Koshigaya Hospital, 2-1-50, Minami Koshigaya, Koshigaya, Saitama 343-8555 Japan; Department of Pathology, Dokkyo Medical University Koshigaya Hospital, 2-1-50, Minami Koshigaya, Koshigaya, Saitama 343-8555 Japan; Department of Medical Examination, Shiba Park Clinic, 2-4-1 Shibakouen, Minato-ku, Tokyo 105-0011 Japan

**Keywords:** Ulcerative colitis, Dysplasia, Carcinoma, Laparoscopic, HALS, IPACA, IPAA, Surveillance

## Abstract

**Background:**

Ulcerative colitis (UC) patients have an increased risk of colorectal dysplasia and carcinoma. The purpose of this study was to analyze the clinical features and surgical treatment of ulcerative colitis associated with dysplasia or carcinoma.

**Methods:**

We operated on 41 UC patients since April 2000. Twelve of the cases were associated with dysplasia or carcinoma. Ten patients were male and two were female; the median age was 58.0 years, and the average duration of disease was 19.2 years. Nine cases were pancolitis type and three were left-sided type. Six cases were remission-relapsing type and six were chronic inflammation type. In 10 of 12 cases, dysplasia or carcinoma was diagnosed before the operations. Nine cases were primary operations and two were second-time operations.

**Results:**

Among ten patients who underwent primary operations, four patients had open surgery and six patients had hand-assisted laparoscopic surgery (HALS). Seven patients received anus/anal sphincter-preserving operations with reconstruction by the ileal pouch technique. Ileal pouch anal-canal anastomosis (IPACA) was performed in five cases and ileal pouch anal anastomosis (IPAA) in two cases. Abdomino-peritoneal resection was performed in two cases, proctcolectomy with permanent ileostomy in one case, and right hemicolectomy in one case. A 39-year-old patient was unresectable due to dissemination of the carcinoma. A 55-year-old patient who underwent IPACA showed night soiling postoperatively. Other patients who received IPAA and IPACA showed favorable anal function postoperatively. Histological examination showed low-grade dysplasia in two cases, high-grade dysplasia in three cases, and adenocarcinoma in seven cases. In the seven cases of adenocarcinoma, four, two, and one cases were stage 1, 3, and 4 according to TNM classification. Three of five cases with dysplasia were detected by surveillance colonoscopy. All patients with carcinoma were symptomatic and did not undergo surveillance colonoscopy.

**Conclusions:**

IPACA by HALS was safely performed as an anal-preserving operation in UC patients with dysplasia or carcinoma. Non-anal-preserving operations for aged patients showed a preferable postoperative course. Surveillance colonoscopy is essential for detecting dysplasia before the development of carcinoma.

## Background

Ulcerative colitis (UC) patients have an increased risk of dysplasia and developing colorectal carcinoma (CRC). Patients with UC have a 2.4-fold increased overall CRC risk [1]. The cumulative probability of UC patients developing CRC is 2% by 10 years, 8% by 20 years, and 18% by 30 years, according to a recent meta-analysis [2].

Chronic inflammation and the increased turnover of epithelial cells contribute to the development of dysplasia which may further transform into CRC [3]. Riddell described two categories of dysplasia associated with UC, *indefinite* and *positive* [4]. The indefinite category includes the subcategories probably negative (probably inflammatory), unknown, and probably positive (probably dysplastic). The positive category includes low-grade dysplasia (LGD) and high-grade dysplasia (HGD). Kiran reported that carcinoma was present in 29% of patients with preoperative HGD compared with 3% in those with LGD [5]. Thus, the risk of carcinoma in patients with HGD is substantial. It is therefore important to conduct surveillance colonoscopy to detect dysplasia before CRC develops [6].

The standard surgical procedure for patients with UC is restorative proctocolectomy with construction of a hand-sewn ileal pouch anal anastomosis (IPAA) or an ileal pouch anal-canal anastomosis (IPACA) created with a circular stapler. IPAA totally removes the rectal mucosa and eliminates the risk of future development of carcinoma in the anorectal cuff. However, the IPAA procedure is more complicated and time-consuming compared with IPACA. The preservation of anal function following IPAA, especially in older patients, is the subject of debate and has been scarcely addressed in the scientific literature. It has been reported that IPAA can be safely offered to selected old UC patients who are strongly motivated and who have no clinical disturbances of continence [7]. Al-Sukhni reported that IPACA does not appear to be inferior to IPAA with respect to oncologic outcomes, and it seems appropriate in patients with UC associated with coexisting dysplasia or carcinoma [8].

Laparoscopic procedures have been applied to total proctocolectomy for UC patients. Compared with open surgery, laparoscopic surgery for UC was shown to be at least as safe and was superior with respect to postoperative fasting time, postoperative hospital stay, and overall complication rate, according to the results of a meta-analysis [9]. However, the clinical value of laparoscopic surgery for UC needs further evaluation. Colectomy for UC patients with dysplasia or carcinoma requires sufficient lymph node resections with mobilization of the mesenteries and high ligation of the arteries. Tsuruta reported that hand-assisted laparoscopic surgery (HALS) had a significantly lower operative time compared with conventional laparoscopic proctocolectomy [10]. In the present study, we performed HALS for proctocolectomy of UC patients. Colectomy was performed in 41 UC patients. Dysplasia or carcinoma was observed in 12 cases, and these cases were analyzed clinicohistopathologically.

## Methods

A total of 41 UC patients underwent colectomy from April 2000 to December 2013. Nineteen patients were male and twenty two were female. The median age was 41.4 years (range 19 to 73).

Histological diagnosis of dysplasia and carcinoma associated with UC was performed according to the criteria described by Riddell [4]. Twelve cases were confirmed to be associated with dysplasia or carcinoma based on histological assessment of resected specimens, and these twelve cases were enrolled in the study. Clinicopathological classifications and stage groupings for carcinoma were performed based on the seventh edition TNM classification of colorectal cancer [11]. Written informed consent was obtained from all patients.

## Results

### Surgical resection procedures

**Figure 1:**
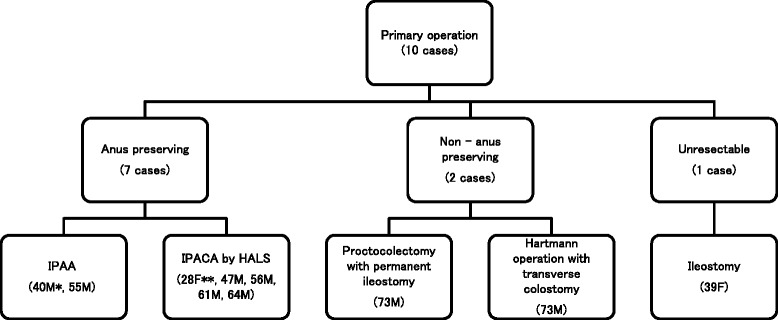
**Surgical procedures for primary resection in ulcerative colitis cases associated with dysplasia or carcinoma.** Asterisk indicates the procedure was done by HALS; double asterisk indicates carcinoma was not diagnosed preoperatively. IPAA = ileal pouch anal anastomosis, IPACA = ileal pouch anal-canal anastomosis, HALS = hand-assisted laparoscopic surgery.

Ten cases were primary operations (Figure 1), and two cases were second-time operations. In seven of ten primary operations, the anus and anal sphincter were preserved and permanent colostomy was avoided. Five IPACA cases underwent HALS. The average operation time for HALS was 310 min. The average bleeding volume was 350.6 g. In one patient in whom IPACA by HALS was started, IPAA was performed instead due to difficulties with the stapling anastomosis procedure. Two patients received permanent stoma. One 39-year-old female patient with carcinoma was unresectable due to peritoneal dissemination and underwent ileostomy instead.

In the two patients who had a second operation, the procedures performed were abdomino-peritoneal dissection and left colectomy.

### Postoperative complications

A 55-year-old male patient who received IPAA showed soiling in the nighttime after ileostomy closure. This led the patient to use a pad while sleeping. A 73-year-old male patient who underwent abdomino-peritoneal resection and transverse colostomy showed prolapse of the stoma 3 months after the original operation. The stoma was subsequently reconstructed.

### Histology

Twelve cases were confirmed to be associated with dysplasia or carcinoma based on histology of the resected specimen. The characteristics of these 12 patients are presented in Table 1. Dysplasia or carcinoma was diagnosed preoperatively in 9 of the 12 cases. Two patients who had a colectomy due to steroid-dependent UC were not diagnosed with dysplasia preoperatively. However, histological examination demonstrated dysplasia in the resected specimen postoperatively. Patients who were diagnosed with dysplasia or carcinoma preoperatively were detected by colonoscopy. Three patients who were diagnosed with dysplasia preoperatively were followed-up by surveillance colonoscopy, while patients with carcinoma did not undergo surveillance colonoscopy (Table 2).

**Table 1:** **Characteristics of ulcerative colitis cases associated with dysplasia or carcinoma**

**Characteristics**	
No. of patients	12
Male/female	10/2
Age (years), median (range)	58.0 (28 to 73)
Average duration of disease (years), median (range)	19.2 (12 to 30)
Site of inflammation	
Pan colitis	9
Left-sided colitis	3
Inflammation type	
Remission-relapsing	6
Chronic inflammation	6
Operation	
Primary operation	10
Reoperation	2
Laparoscopic surgery	
Open surgery	6
HALS	6
Preoperative diagnosis of dysplasia or carcinoma	
Yes	10
No	2

*HALS* hand-assisted laparoscopic surgery.

**Table 2:** **Reasons for surgical resection in UC cases associated with dysplasia or carcinoma**

	**Dysplasia (** ***n*** **= 5)**	**Carcinoma (** ***n*** **= 7)**
Steroid dependent	2	0
Stenosis	0	1
Preoperative diagnosis of dysplasia or carcinoma by colonoscopy	3	6

The grade of dysplasia and TNM staging of carcinomas are presented in Table 3. Eight patients showed single lesion of dysplasia or carcinoma in the resected specimen. Two patients with low-grade or high-grade dysplasia showed double lesions of dysplasia in the resected specimens. Two patients with stage II or III carcinoma showed triple carcinomas in the resected specimens.

**Table 3:** **Grade of dysplasia and TNM staging of carcinoma**

**TNM**	**Histological type**	**No. of patients with single lesion**	**No. of patients with multiple lesions**
	Low-grade dysplasia	1	1
	High-grade dysplasia	2	1
pT1N0M0	Well-differentiated adenocarcinoma	3	0
pT2N0M0	Well-differentiated adenocarcinoma	0	1
pT3N1M0	Well-differentiated adenocarcinoma	1	1
sT4NXM1	Well-differentiated adenocarcinoma	1	0
		8	4

## Discussion

We performed colectomies in 12 UC patients with dysplasia or carcinoma. In six patients, proctocolectomy was performed by HALS, which was associated with a shorter operation time compared with conventional laparoscopic proctocolectomy. Although HALS is a useful procedure, the length of incision is longer than that with conventional laparoscopic surgery. Recently, we began performing pure laparoscopic proctocolectomy without lymph node resection for UC patients who do not have a preoperative diagnosis of dysplasia or carcinoma.

We performed IPACA mainly for reconstruction after proctocolectomy because the IPAA procedure is more complicated and time-consuming than IPACA. A 55-year-old patient who received IPAA showed night soiling after the operation. With respect to preservation of anal function, IPAA may not be suitable for patients older than middle age. IPACA also carries the risk of carcinoma in the remnant rectal mucosa. The risk is quite small, however, and colonoscopy surveillance should be performed to monitor the remaining rectal mucosa.

The number of UC patients is increasing in Japan as well as in Western countries, and the number of UC patients with a long duration of disease is also rising. When UC patients reach middle age, the risk of dysplasia and carcinoma increases. The rate of dysplasia or carcinoma among operated UC patients is increasing in our hospital (data not shown), which is a disturbing trend. Surveillance colonoscopy for dysplasia and carcinoma is recommended for patients with long-standing UC. Patients in whom dysplasia or carcinoma is detected by surveillance colonoscopy show a better prognosis, while those who do not undergo surveillance often develop advanced carcinoma and have a poor prognosis [12]. We performed colectomy in a total of 41 UC patients; 12 patients showed dysplasia or carcinoma, and 3 of 5 dysplasia cases in patients were detected by surveillance colonoscopy. None of the patients with carcinoma underwent surveillance colonoscopy. These patients, upon presentation of symptoms, underwent colonoscopy, which underscores the importance of surveillance colonoscopy. Two patients who had colectomies due to steroid-dependent UC were not diagnosed with dysplasia preoperatively but were diagnosed based on analysis of resected specimens postoperatively. In such cases, endoscopic detection of dysplasia might be difficult because of mucosal inflammation. It is reported that CRC-associated UC showed multiple lesions (11%) [13]. In our study, two of seven patients with carcinoma showed the triple carcinoma lesions.

Advanced carcinoma affects the lifespan of UC patients. In a study conducted in Japan, Watanabe reported that patients with CRC-associated UC showed poorer survival rates than those with sporadic CRC in the advanced stage, while no difference was observed in the early stage [13]. It is important to detect dysplasia and carcinoma in an early stage by surveillance, and surveillance colonoscopy for UC patients with long disease duration is essential.

## Conclusions

IPACA by HALS was safely performed as an anal-preserving operation in UC patients with dysplasia or carcinoma. Non-anal-preserving operations for aged patients showed a preferable postoperative course. Surveillance colonoscopy for UC patients with long disease duration is essential for detecting dysplasia before the development of carcinoma.

## Abbreviations

CRC: Colorectal carcinoma
HALS: Hand-assisted laparoscopic surgery
HGD: High-grade dysplasia
IPAA: ileal pouch anal anastomosis
IPACA: ileal pouch anal-canal anastomosis
LGD: Low-grade dysplasia
UC: Ulcerative colitis
